# Longitudinal Changes and Recovery in Heart Rate Variability of Young Healthy Subjects When Exposure to a Hypobaric Hypoxic Environment

**DOI:** 10.3389/fphys.2021.688921

**Published:** 2022-01-13

**Authors:** Chenbin Ma, Haoran Xu, Muyang Yan, Jie Huang, Wei Yan, Ke Lan, Jing Wang, Zhengbo Zhang

**Affiliations:** ^1^Center for Artificial Intelligence in Medicine, Medical Innovation Research Department, PLA General Hospital, Beijing, China; ^2^Beijing Advanced Innovation Center for Biomedical Engineering, Beihang University, Beijing, China; ^3^School of Biological Science and Medical Engineering, Beihang University, Beijing, China; ^4^Shenyuan Honors College, Beihang University, Beijing, China; ^5^Medical School of Chinese PLA, Beijing, China; ^6^Department of Hyperbaric Oxygen Therapy, The First Medical Center, Chinese PLA General Hospital, Beijing, China; ^7^Beijing SensEcho Science & Technology Co., Ltd., Beijing, China; ^8^School of Computer and Information Technology, Beijing Jiaotong University, Beijing, China

**Keywords:** heart rate variability, multiscale entropy, acute exposure, autonomic nervous system, longitudinal change

## Abstract

**Background:** The autonomic nervous system (ANS) is crucial for acclimatization. Investigating the responses of acute exposure to a hypoxic environment may provide some knowledge of the cardiopulmonary system’s adjustment mechanism.

**Objective**: The present study investigates the longitudinal changes and recovery in heart rate variability (HRV) in a young healthy population when exposed to a simulated plateau environment.

**Methods**: The study followed a strict experimental paradigm in which physiological signals were collected from 33 healthy college students (26 ± 2 years, 171 cm ± 7 cm, 64 ± 11 kg) using a medical-grade wearable device. The subjects were asked to sit in normoxic (approximately 101 kPa) and hypoxic (4,000 m above sea level, about 62 kPa) environments. The whole experimental process was divided into four stable resting measurement segments in chronological order to analyze the longitudinal changes of physical stress and recovery phases. Seventy-six time-domain, frequency-domain, and non-linear indicators characterizing rhythm variability were analyzed in the four groups.

**Results:** Compared to normobaric normoxia, participants in hypobaric hypoxia had significantly lower HRV time-domain metrics, such as RMSSD, MeanNN, and MedianNN (*p* < 0.01), substantially higher frequency domain metrics such as LF/HF ratio (*p* < 0.05), significantly lower Poincaré plot parameters such as SD1/SD2 ratio and other Poincaré plot parameters are reduced considerably (*p* < 0.01), and Refined Composite Multi-Scale Entropy (RCMSE) curves are reduced significantly (*p* < 0.01).

**Conclusion:** The present study shows that elevated heart rates, sympathetic activation, and reduced overall complexity were observed in healthy subjects exposed to a hypobaric and hypoxic environment. Moreover, the results indicated that Multiscale Entropy (MSE) analysis of RR interval series could characterize the degree of minor physiological changes. This novel index of HRV can better explain changes in the human ANS.

## Introduction

As is well known, acute exposure to a plateau environment tends to activate a series of stress responses. Typically, it manifests headache, insomnia, appetite loss, dyspnea, or even can be developed as acute mountain sickness. Evidence suggests that this acute hypoxia may cause systemic metabolic imbalances and increase cardiovascular disease risks (Riley and Gavin, 2017; Parati et al., 2018). For example, sudden cardiac death makes up 30% of all mountain sports deaths at altitude (Burtscher, 2007). Furthermore, hypoxia can induce tachycardia when the concentration of oxygen is lower than 17% (Iwasaki et al., 2006), and the risk of cardiac arrhythmia is significantly increased in healthy older populations when exposed to moderate altitude (Kujaník et al., 2000).

Researchers are of great interest in the stress response process during acute adaptation to a hypobaric hypoxic environment in the human body, whereas the autonomic nervous system (ANS) is crucial for acclimatization. Acute hypoxia tends to activate sympathetic mechanisms regulating the cardiovascular system, such as increased heart rate, cardiac output, and blood pressure (Perini et al., 1996; Vigo et al., 2010), and may also cause hyperventilation of the respiratory system (Woods et al., 2008). The analysis of HRV is shown to be an effective and non-invasive method to characterize the two branches of the cardiac ANS activity (Malliani et al., 1991; Wille et al., 2012). Particularly, linear parameters of HRV (both time and frequency domain metrics) have been widely used for hypoxia because the results of linear metrics are easy to interpret in physiological terms. Most studies have shown that LF and HF power decreases at high altitudes (Hughson et al., 1994; Perini et al., 1996; Kanai et al., 2001; Sevre et al., 2001; Roche et al., 2002; Cornolo et al., 2004; Saito et al., 2005; Chen et al., 2008; Faini et al., 2019). However, it was also found that a decrease in HF power is accompanied by an increase in LF power (Hughson et al., 1994), or no change in HF power but an increase in LF power (Iwasaki et al., 2006; Boos et al., 2018). Previous studies’ results are inconsistent, attributed to over-complicated sinus rhythm fluctuations and multiple feedback control in the ANS regulation.

Simultaneously, specific non-linear metrics have been shown to reflect the cardiovascular and respiratory systems (Toichi et al., 1997; Lewis and Short, 2007; Zhang et al., 2014; Costa et al., 2017; Yan et al., 2017). We consider that the non-linear methods may produce extra information and analysis perspectives to explore further and interpret the ANS regulation. Therefore, it is necessary to combine time, frequency, and non-linear analysis approaches to dissect heartbeat sequences to characterize longitudinal changes in the ANS mechanism of cardiovascular during acute exposure to hypoxia. The approximate entropy (ApEn) and sample entropy (SampEn) have been investigated to quantize the complexity or irregularity of RR interval series in this scenario (Zhang et al., 2014). Meanwhile, Multiscale Entropy (MSE) analysis can further quantify the irregularity in a time series across multiple scales. Nonetheless, the interpretation of the non-linear results, such as entropy-related results, is yet to be explained largely because few studies have addressed non-linear parameters of cardiovascular changes during the acute hypoxic environment.

Although several studies of the stress response during acute hypoxia have been done in the past 2 decades, we still consider that some limitations remain unsolved. First, most studies concentrate on the variation of HRV during the “normxia-hypoxia” process, while studies on the complete longitudinal changes (i.e., “normxia-hypoxia-normxia”) are still lacking. This means there is also a lack of awareness about the cardiovascular recovery response after hypoxia. On the other hand, as the above statements, we consider that in previous studies, the analysis of HRV was not sufficiently comprehensive, and non-linear methods deserve a thorough study. The present study investigates the longitudinal changes and recovery situations in ANS regulation of healthy young adult subjects during acute hypoxia. Therefore, we have designed an experiment that simulates the hypobaric and hypoxic environment of 4,000 m above sea level (asl), then analyzes the changes of the time and frequency domain HRV features and non-linear RR interval series parameters when acute exposure to this environment.

## Materials and Methods

### Subjects and Experimental Protocol

Thirty-three healthy university students (26 ± 2 years, 171 cm ± 7 cm, 64 ± 11 kg) participated in a simulated altitude oxygen chamber experiment to study the changes in HRV. Table 1 shows the detailed basic demographic information table for the simulated plateau experiment. Healthy populations enter the hypobaric chamber (an enclosed compartment with a suction pump), which exposes them acutely to a hypobaric and hypoxic environment at 4,000 m asl, resulting in stress response and monitoring their state of autonomous recovery. None of the volunteers had reached a height of 2,000 m asl before the experiment’s 6 months. All were asked to avoid drinking alcohol or consuming caffeine 12 h before the start of the investigation. Participants were asked to fill in questionnaires (Self-Rating Scale of Sleep and Self-Rating Anxiety Scale) to assess their health states. All of them did not report any medical or altitude-related problems.

**Table 1:** Basic information table of volunteers for the simulated plateau experiment.

Gender	Male (*n* = 21)	Female (*n* = 12)
**Demographic (mean ± SD)**		
Age	27.33 ± 3.10	25.67 ± 1.72
Height (cm)	175.19 ± 4.61	164.58 ± 7.67
Weight (kg)	71.76 ± 7.73	53 ± 6.21
BMI^a^	23.37 ± 2.79	19.56 ± 1.88
**Scales (mean ± SD)**		
SRSS^b^	17.81 ± 3.36	20.33 ± 4.66
SAS^c^	25.43 ± 4.19	26.92 ± 4.27
Before the experiment, MAP^d^, mmHg, (mean ± SD)	90.48 ± 6.94	84.50 ± 8.17
After the experiment, MAP^d^, mmHg, (mean ± SD)	90.76 ± 8.20	79.81 ± 12.53

a Body Mass Index.

b Self-Rating Scale of Sleep.

c Self-Rating Anxiety Scale.

d Mean Arterial Pressure.

Subjects under health screening sat outside the hyperbaric chamber (sea level) for 30 min to measure their baseline level information, after which they entered the simulated plateau environment. The oxygen chamber was altitude ascended for approximately 20 min (3 m/s), maintained at a low pressure of 4,000 m asl for 1 h, and then the reduction process (return to normoxia) lasted for approximately 20 min. During the simulated plateau experiment, the subjects were asked to sit still inside the chamber. At the end of the experiment, the volunteers were asked to sit still for another 30 min in normoxia to monitor the changes in their HRV (recovery state).

**Figure 1:**
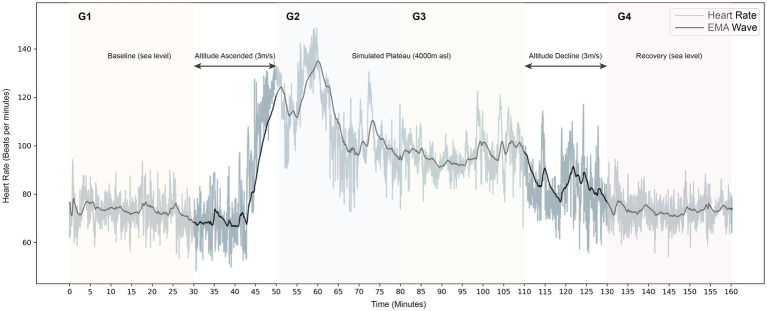
Longitudinal heart rate tachogram is shown in grey, and the exponential smoothing averaging (EMA) is black.

In this study, participants remain awake and resting throughout in different environments of normoxia (approximately 101 kPa) and hypoxia (4,000 m asl, about 62 kPa) and were divided into four steady states in chronological order (G1–G4). The experiment supervisor accompanied and observed the participants throughout the experiment to remain awake and safe in the chamber. To ensure that the data were studied in the resting state, the experimental analysis phase intercepted each steady-state’s middle 21-min period. It is divided into four groups to analyze the longitudinal changes of the physical stress response accordingly. G1 represents the 21-min measurement phase at baseline, and G4 represents the 21-min measurement phase after the simulated plateau experiment. In the controlled hypobaric and hypoxia environment, G2 indicates the first half-hour of the measurement period, while G3 indicates the second half-hour. The experimental flow is shown in Figure 1.

### Measurements and Preprocessing

The subject’s physiological signals were recorded simultaneously through a medical-grade wearable monitoring device, SensEcho (Beijing SensEcho Science & Technology Co., Ltd.), which was developed by our team and has received certification from the China Food and Drug Administration (CFDA; Xu et al., 2020). SensEcho can collect signals including ECG in 200 Hz sample rate, respiratory waves from the chest and abdomen in 25 Hz and triaxial acceleration signals in 25 Hz. In this study, the ECG of the patients were recorded by SensEcho for consecutive 4-h in the longitudinal periods before and after the hyperbaric oxygen chamber experiment.

In this study, the resting ECG signals of each state were intercepted to calculate HRV metrics. To compare the longitudinal change and recovery of physiological parameters, in this study, we divided the experimental process into four 30-min resting segments (G1, G2, G3, and G4) to calculate 76 different HRV time domains, frequency domain, and non-linear indicators, respectively as shown in Table 2.

**Table 2:** HRV metrics analyzed in this study.

**Time-domain**	
RMSSD (ms)	Root mean square of successive RR interval differences
MeanNN (ms)	Mean of NN
SDNN (ms)	SD of NN
SDSD (ms)	SD of the successive differences between NN
CVNN (%)	SDNN divided by MeanNN
CVSD (%)	RMSSD divided MeanNN
MedianNN (ms)	Median of NN
MadNN (ms)	Median absolute deviation of NN
MCVNN (ms)	MadNN divided by MedianNN
IQRNN (ms)	Interquartile range of NN
pNN50 (%)	Percentage of successive NN that differ by more than 50 ms
pNN20 (%)	Percentage of successive NN that differ by more than 20 ms
TINN	Baseline width of the NN distribution obtained by triangular interpolation
HTI	Total number of NN divided by the height of the NN histogram
**Frequency domain**	
LF (ms^2^)	Spectral power density pertaining to low-frequency band (0.04–0.15 Hz)
HF (ms^2^)	Spectral power density pertaining to high-frequency band (0.15–0.4 Hz)
VHF (ms^2^)	Absolute power of the very-high-frequency band (0.4–0.5 Hz)
LF/HF ratio	Ratio of LF-to-HF power
LFn (n.u.)	The normalized low frequency, low-frequency power divided by the total power
HFn (n.u.)	The normalized high frequency, high-frequency power divided by the total power
LnHF (ms^2^)	The log-transformed HF
**Non-linear metrics**	
**Characteristics of the Poincaré Plot Geometry**	
SD1	Index of short-term NN fluctuation, the semi-short axis of the fitted ellipse in the Poincaré plot
SD2	Index of long-term NN fluctuation, the semi-long axis of the fitted ellipse in the Poincaré plot
SD1/SD2 ratio	Ratio of SD1-to-SD2
S	Area of the fitted ellipse in the Poincaré plot
CSI	Cardiac Sympathetic Index
CVI	Cardiac Vagal Index
MCSI	Modified CSI
**Indices of Heart Rate Fragmentation**	
PIP	Percentage of inflection points of the RR intervals series
IALS	Inverse of the average length of the acceleration/deceleration segments
PSS	Percentage of short segments
PAS	IPercentage of NN intervals in alternation segments
**Indices of Heart Rate Asymmetry (HRA)**	
GI	Guzik’s Index
SI	Slope Index
AI	Area Index
PI	Porta’s Index
C1d	Contributions of heart rate decelerations to short-term HRV
C1a	Contributions of heart rate accelerations to short-term HRV
SD1d	Short-term variance of contributions of decelerations (prolongations of RR intervals)
SD1a	Short-term variance of contributions of accelerations (shortenings of RR intervals)
C2d	Contributions of heart rate decelerations to long-term HRV
C2a	Contributions of heart rate accelerations to long-term HRV
SD2d	Long-term variance of contributions of decelerations
SD2a	Long-term variance of contributions of accelerations
Cd	Total contributions of heart rate decelerations to HRV
Ca	Total contributions of heart rate accelerations to HRV
SDNNd	Total variance of contributions of decelerations
SDNNa	Total variance of contributions of accelerations
**Indices of Complexity**	
ApEn	Approximate entropy (ApEn) of HRV
SampEn	Sample entropy of HRV
Slope 5	Linear-fitted slope between complexity index (CI) 1–5
Area 1–5	Area under refined composite multiscale entropy (RCMSE) curve between CI 1–5
Area 6–15	Area under RCMSE curve between CI 6–15
Area 6–20	Area under RCMSE curve between CI 6–20
**Power-law analysis**	
DFA	Scale index of detrended fluctuation analysis
MFDFA	Scale index of multifractal DFA

### HRV Metrics

In a standard ECG, the RR interval is defined as the R wave on two adjacent regular QRS complex waves. The HRV is represented by a continuous RR interval, defined as the Normal-to-Normal interval (NN). The time-domain analysis methods of HRV are divided into statistical and geometric methods according to the presentation. The former process involves subjecting the NN to various statistically relevant calculations of variability size, such as the overall variability of the regular sinus interval expressed by metrics such as SDNN, CVNN, etc., where SDANN relates to the variance below the 0.0033 Hz spectral frequency. The geometric method focuses on the probability distribution of NN by plotting histograms of RR interval differences and obtaining quantitative time-frequency parameters based on the graphs, mainly including TINN and HTI, which are used to assess the overall HRV level.

The time-domain features can demonstrate the waveform variation with time. Still, the time-domain parameters are more difficult for higher-order systems to determine the signal transients and are more affected by non-stationarity. Power spectral density (PSD) is often used to demonstrate ECG signals’ distribution and phase variation across frequency bands. Frequency-domain measurements estimate the distribution of absolute or relative power at four frequency bands through fast Fourier transform. We divided heart rate oscillations into ULF, VLF, LF, HF, and VHF bands (see Table 2). ULF and VLF usually require long-term recordings of Holter monitoring and thus were not included in this study. These features are generally interpreted as markers of the integral sympathovagal balance.

The concept of nonlinearity and chaos is a fusion of topology and differential equation theory, which characterizes the heartbeat activity state’s qualitative and quantitative variation patterns and explores the ANS’s complexity. Many studies have confirmed that the non-linear properties of HRV mainly include chaos and fractals, i.e., irregularities and self-similar structures of heartbeats in a deterministic system (Vigo et al., 2010; Porta et al., 2012, 2015; Zhang et al., 2015). Non-linear analysis methods are currently divided into graphical and parametric algorithms. Refined Composite Multi-Scale Entropy (RCMSE) addresses the drawback that SampEn does not give accurate complexity estimates at large scales while reducing the probability of inducing fuzzy entropy for shorter time series (Wu et al., 2014). This study mainly investigates the Poincaré plot, detrended fluctuation analysis (DFA), and Entropy (ApEn, SampEn, and RCMSE).

### Statistical Analysis

RStudio (version 1.4.1103) was used for statistical analysis in this study. The primary purpose of statistical analysis is to determine whether there are significant differences between specific groups. The Shapiro-Wilk test is used to determine whether the calculated characteristic distribution obeys the assumption of normality. The one-way repeated measures ANOVA test was performed on multiple samples if the variable’s distribution followed a normal distribution. The greenhouse-Geisser correction was performed for instances that violated the spherical assumption condition in Mauchly’s Test. Numerous comparisons between two sets of samples were performed using the Bonferroni *post hoc* test for paired *t*-tests. Otherwise, we used the non-parametric two-way ANOVA by ranks test for multiple sample correlation if the data distribution did not satisfy the normality assumption. Specifically, the Friedman test was chosen when samples were matched for testing between-group differences. The Bonferroni-adjusted *p* values (significant level = 0.05) were used in multiple comparisons.

## Results

A typical example of the heart rate dynamic fluctuations during the experiment is shown in Figure 1. It is visible that there is a strong increment in heart rate during the stable plateau environment (4,000 asl) compared to the baseline. In addition, intuitively, the patterns of heart rate fluctuations show great homogeneity during the baseline period (G1) and the recovery period (G4).

### Time Domain

**Figure 2:**
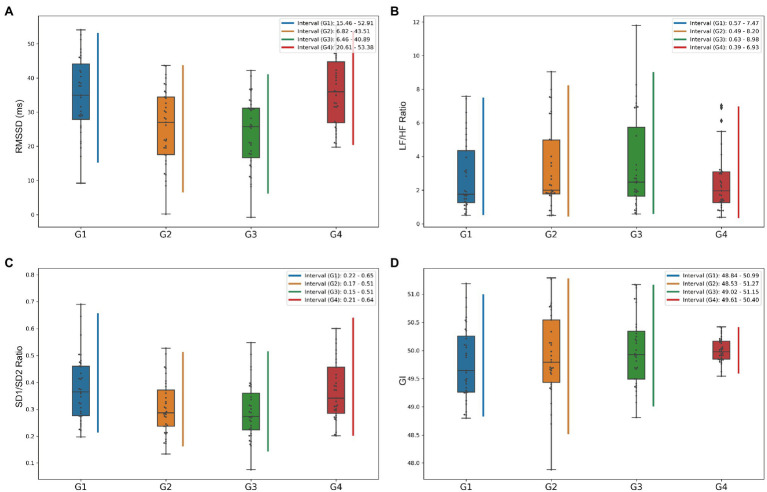
Box plots of heart rate variability (HRV) metrics to show the variation of data distribution by longitudinal grouping. **(A–D)** Shows box plots of the distributions of RMSSD, LF/HF ratio, SD1/SD2 ratio, and GI indices, respectively, and the vertical lines represent the central 95% intervals of the distributions of each group.

Heart rate variability time-domain metrics for each group measured longitudinally are presented in Table 3. As expected, the subjects showed significant changes in most HRV time-domain parameters compared to the baseline state in the simulated plateau’s experimental setting. The RMSSD, reflecting the level of parasympathetic activity (Ciccone et al., 2017), was the most significant between-group difference among the groups, with significant differences in multiple longitudinally varying states (*p* < 0.001). The state of the subjects in the simulated plateau experiment was significantly different compared to the period outside the oxygen chamber (as shown in Figure 2A). The calculation of RMSSD was more stable compared to pNN50, which usually reflects abrupt changes in the RR interval and is also a high-frequency component of heart rate variability (HRV; Ciccone et al., 2017); however, as shown in Table 3, there was no significant difference in pNN50 changes (*p* = 0.816). Similar to pNN50, SDSD, CVNN, and CVSD reflect the high-frequency variance components of heart rate estimated from short time course measurements (Stein, 2002; Shaffer and Ginsberg, 2017), were positively correlated with each other. And calculations showed that these HRV characteristics were significantly different from baseline (*p* < 0.001) when subjects were in a simulated plateau environment.

**Table 3:** HRV time-domain features (absolute values are means ± SD).

	G1	G2	G3	G4
RMSSD (ms)	34.9 ± 11.2	25.8 ± 11.1^***^	24.1 ± 10.4^***^^,^^###^	35.9 ± 10.2^###^^,^^†††^
MeanNN (ms)	789.8 ± 111.7	758.3 ± 111.0^***^	756.9 ± 110.7^***^	793.8 ± 103.1
SDNN (ms)	55.0 ± 17.6	57.5 ± 18.4^*^	57.8 ± 17.1^*^	60.5 ± 16.8
SDSD (ms)	34.8 ± 10.6	39.1 ± 12.1^***^	41.7 ± 11.7^***^^,^^###^	41.8 ± 18.4
CVNN (ms)	0.070 ± 0.021	0.075 ± 0.021^***^	0.076 ± 0.020^***^	0.076 ± 0.017
CVSD (ms)	0.045 ± 0.014	0.050 ± 0.014^***^	0.053 ± 0.014^***^^,^^###^	0.048 ± 0.016
MedianNN (ms)	785.1 ± 113.3	754.3 ± 113.1^***^	763.5 ± 114.0^***^^,^^###^	799.0 ± 114.8
MadNN (ms)	52.1 ± 16.8	45.1 ± 14.5^***^	47.0 ± 14.7^**^^,^^#^	54.4 ± 13.9
MCVNN (ms)	0.066 ± 0.020	0.059 ± 0.019	0.060 ± 0.019	0.072 ± 0.021
IQRNN (ms)	71.6 ± 22.5	66.3 ± 21.1^*^	63.5 ± 20.4^***^	73.6 ± 17.9
pNN50 (%)	12.30 ± 9.64	13.33 ± 10.57	14.00 ± 12.36	14.57 ± 12.11
pNN20 (%)	43.9 ± 17.8	45.5 ± 19.7	44.9 ± 19.6	44.9 ± 18.1
TINN	289.0 ± 91.3	270.1 ± 94.9	278.7 ± 96.7	321.7 ± 71.9
HTI	5.3 ± 0.5	5.1 ± 0.6	5.2 ± 0.5	5.2 ± 0.3

Physiological variables (*N* = 33). G1, baseline; G2, early simulated plateau; G3, later simulated plateau; and G4, recovery.

* *p* < 0.05;

** *p* = 0.002;

*** *p* < 0.001 for difference with G1.

# *p* < 0.05;

### *p* < 0.001 for difference with G2.

††† *p* < 0.001 for difference with G3.

Besides, MeanNN, MedianNN, and MadNN are usually chosen to assess the fluctuation of heart rate. As shown in Table 3, the hypobaric and hypoxic environment subjects showed an increase in heart rate compared to the baseline level, and the degree of change was significantly different. Still, the recovery status after the simulated plateau experiment was not significantly different from the baseline. SDNN generally reflects the overall level of HRV and incorporates the various frequency components of variability. SDNN showed increased overall variability in the first half-hour (*p* = 0.042) and second half-hour (*p* = 0.046) of the plateau simulation experiment compared to the baseline state. Notably, neither the geometric method parameters TINN nor HTI, which assess the overall level of HRV, were significantly different.

### Frequency Domain

**Figure 3:**
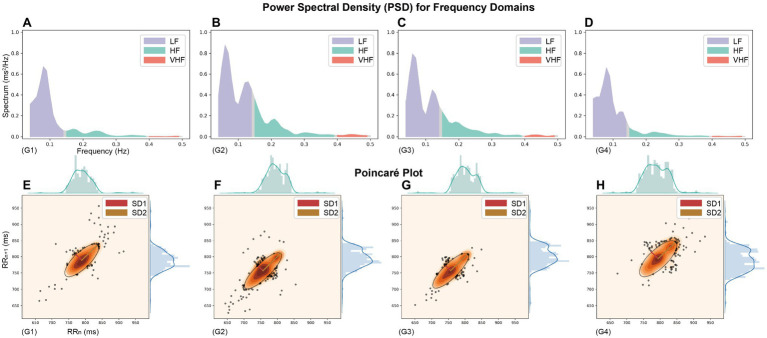
The Power spectral density (PSD) and Poincaré plot throughout the simulated plateau experiment (Heart rate recording data from a randomly selected participant). **(A–D)** PSD for frequency domains and **(E–H)** Poincaré Plot Geometry during G1–G4 phases, the images are plotted on the same scale as the axes chosen to visualize the vertical changes.

It is now generally accepted that the HF component of HRV mainly reflects the level of parasympathetic activity. In contrast, the LF component primarily reflects the joint sympathetic and parasympathetic activity levels. LF/HF ratio is used to measure the balance between sympathetic and parasympathetic nerves (Boardman et al., 2002; Bachler, 2017). The frequency-domain characteristics of HRV are shown in Table 4 and Figures 3A–D. LF metrics increased significantly from baseline levels longitudinally over time, and the LF/HF ratio within the simulated plateau experiment also changed significantly compared to baseline. Still, equilibrium was restored more quickly after the investigation. Also, the standardized LF and HF indicators reflecting sympathetic and parasympathetic regulation changes did not differ (*p* = 0.784, *p* = 0.636, for LFn and HFn, respectively). The box plot in Figure 2B shows that the LF/HF ratio in the G4 phase, which indicates the recovery state after the simulated plateau experiment, was more concentrated relative to the distribution in the investigation.

**Table 4:** HRV frequency domain features (absolute values are means ± SD).

	G1	G2	G3	G4
LF (ms^2^)	1,888.4 ± 1010.1	2,323.3 ± 1,269.9	2,297.9 ± 955.4^***^^,^^##^	2,400.3 ± 909.0^†††^
HF (ms^2^)	983.7 ± 717.0	1,159.0 ± 870.4	1,146.1 ± 907.0	1,560.9 ± 1,173.5
VHF (ms^2^)	85.2 ± 61.1	92.0 ± 64.2	90.2 ± 64.7	107.2 ± 58.7
LF/HF ratio	2.83 ± 2.11	3.25 ± 2.49^**^	3.53 ± 2.82^**^	2.48 ± 1.82
LFn (n.u.)	62.9 ± 17.4	62.5 ± 18.8	63.7 ± 18.4	62.4 ± 14.9
HFn (n.u.)	32.7 ± 15.6	32.9 ± 16.2	31.6 ± 15.9	34.0 ± 14.0
LnHF (ms^2^)	4.85 ± 0.85	4.76 ± 0.91	4.80 ± 0.90	4.68 ± 0.75

Physiological variables (*N* = 33). G1, baseline; G2, early simulated plateau; G3, later simulated plateau; and G4, recovery.

** *p* = 0.002;

*** *p* < 0.001 for difference with G1.

## *p* < 0.01.

††† *p* < 0.001 for difference with G3.

### Non-Iinear Analysis

In this study, using classical chaos theory, power-law analysis, and information-theory-based methods, non-linear metrics of multiple dimensions were calculated to analyze further numerous independent factors of coordinated control of cardiac electrical activity. The non-linear characteristics presented in Table 4 and Figures 3E–H show that the measured parameters related to the Poincaré plot, calculated based on classical chaos theory, are significantly different from baseline. And SD1 characterizes parasympathetic activity, with the cardiac sympathetic index (CSI) considerably lower in subjects exposed to intense pressure and hypoxia than baseline levels. However, the change in SD2, which indicates the degree of overall HRV (Toichi et al., 1997), was not significant (G3 decreased by 7.99 relative to G1, *p* = 0.018). Taken together, the box plots in Figure 2C show that the SD1/SD2 ratio for the simulated oxygen chamber experiment decreased relative to baseline and post-trial recovery status. In addition, the Poincaré Plot Geometry during the G1–G4 phases demonstrated by Figures 3E–H reveals that subjects are more prone to arrhythmias in a hypobaric and hypoxic environment.

This study also characterized the prevalence of heart rate asymmetry (HRA) in the healthy heart physiological system using parameters such as Guzik’s index (GI), Slope index (SI), area index (AI), and Porta’s index (PI; Yan et al., 2017). The combined Figure 2D shows that the distribution of G4 was more concentrated, but the distribution did not differ between groups, indicating that there was no significant change in physiological status.

**Figure 4:**
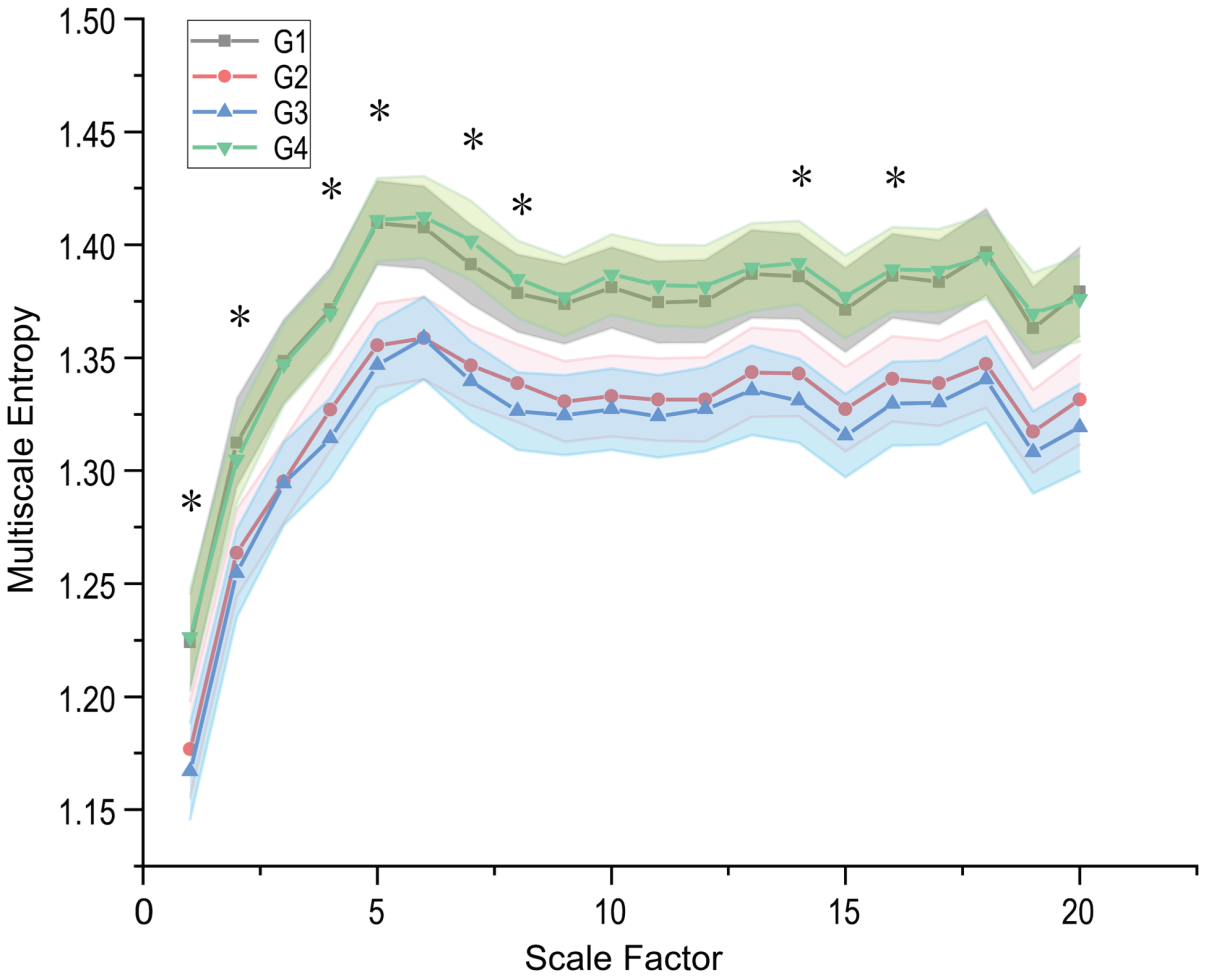
Refined Composite Multi-Scale Entropy (RCMSE) curves (mean ± SE) for participants in the plateau simulation experiment. G1–G4 represents the four steady states in the experiment, respectively, and star-labelled complex indices represent significant differences in G2 and G3 (value of *p* < 0.05). The differences in complexity index (CI) of RCMSE with longitudinal changes over time are mainly concentrated in the low-frequency band (smaller scales).

Besides, heart rate fragmentation [PIP, Inverse of the average length of the acceleration/deceleration segments (IALS), Percentage of short segments (PSS), and IPercentage of NN intervals in alternation segments (PAS); Costa et al., 2017] did not show significant differences. In this paper, the entropy-based approach investigates the predictability (amount or increment of information), complexity, and other characteristics of heart rate control systems. The ApEn and SampEn quantified the incremental data of increasing coding length, but the sequence complexity demonstrated in Table 5 did not change. As shown in Figure 4, the RCMSE was calculated by subjecting the original sequences to complexity measures at different scales and found that the area under the curve for subjects under the simulated plateau implementation with complexity indices 6–15 decreases relatively compared to the baseline (*p* = 0.022 and *p* = 0.010 for G1–G2 and G1–G3, respectively).

**Table 5:** HRV non-linear features (absolute values are means ± SD).

	G1	G2	G3	G4
SD1	24.4 ± 7.7	21.6 ± 8.1^***^	21.6 ± 8.0^***^	27.6 ± 10.1
SD2	66.8 ± 18.5	72.0 ± 21.2	74.8 ± 19.9^*^	73.3 ± 12.8
SD1/SD2 ratio	0.38 ± 0.12	0.30 ± 0.10^***^	0.29 ± 0.11^***^	0.38 ± 0.13
S	5,324.8 ± 2,391.9	5,130.9 ± 2,714.3	5,355.2 ± 2,871.5	6,516.0 ± 2,976.6
CSI	2.91 ± 0.92	3.61 ± 1.14^***^	3.72 ± 1.17^***^	2.93 ± 0.92^†^
CVI	4.46 ± 0.26	4.43 ± 0.25	4.43 ± 0.25	4.43 ± 0.27
MCSI	958.4 ± 489.0	930.6 ± 469.4	927.8 ± 459.2	986.9 ± 335.0
PIP	0.57 ± 0.06	0.57 ± 0.07	0.57 ± 0.07	0.56 ± 0.05
IALS	0.56 ± 0.07	0.55 ± 0.07	0.55 ± 0.07	0.54 ± 0.06
PSS	0.80 ± 0.10	0.79 ± 0.10	0.79 ± 0.10	0.79 ± 0.08
PAS	0.15 ± 0.09	0.15 ± 0.10	0.15 ± 0.09	0.14 ± 0.07
GI	49.78 ± 0.65	49.90 ± 0.75	49.99 ± 0.64	50.01 ± 0.21
SI	49.70 ± 0.71	49.91 ± 0.65	50.01 ± 0.62	50.01 ± 0.20
AI	49.82 ± 0.66	49.90 ± 0.73	50.00 ± 0.71	50.02 ± 0.22
PI	47.73 ± 3.75	47.72 ± 3.85	47.79 ± 3.88	47.06 ± 2.58
C1d	0.48 ± 0.06	0.48 ± 0.06	0.48 ± 0.05	0.48 ± 0.04
C1a	0.52 ± 0.06	0.52 ± 0.06	0.52 ± 0.05	0.52 ± 0.04
SD1d	16.93 ± 5.74	16.88 ± 5.43	17.02 ± 5.63	18.35 ± 6.44
SD1a	17.73 ± 5.78	17.72 ± 5.54	17.05 ± 5.55	19.27 ± 6.81
C2d	0.51 ± 0.06	0.51 ± 0.06	0.51 ± 0.07	0.52 ± 0.03
C2a	0.49 ± 0.06	0.49 ± 0.06	0.49 ± 0.07	0.48 ± 0.03
SD2d	51.46 ± 16.05	53.04 ± 17.68	52.87 ± 17.49	57.92 ± 17.03
SD2a	50.07 ± 17.08	51.81 ± 19.27	51.83 ± 19.43	56.26 ± 16.76
Cd	0.51 ± 0.04	0.51 ± 0.05	0.51 ± 0.05	0.51 ± 0.02
Ca	0.49 ± 0.04	0.49 ± 0.05	0.49 ± 0.05	0.49 ± 0.02
SDNNd	38.84 ± 11.94	38.28 ± 11.37	37.22 ± 10.35	40.23 ± 8.13
SDNNa	38.36 ± 13.03	38.12 ± 12.74	36.41 ± 10.73	39.64 ± 7.19
ApEn	0.96 ± 0.08	0.94 ± 0.08	0.94 ± 0.09	0.93 ± 0.04
SampEn	1.39 ± 0.34	1.33 ± 0.34	1.40 ± 0.25	1.41 ± 0.19
Slope 5	0.05 ± 0.07	0.04 ± 0.07	0.04 ± 0.07	0.05 ± 0.07
Area 1-5	5.35 ± 1.24	5.35 ± 1.24	5.36 ± 1.24	5.34 ± 1.22
Area 6-15	11.04 ± 2.48	11.09 ± 2.50^*^	11.10 ± 2.48^*^	11.08 ± 2.46
Area 6–20	17.94 ± 4.05	18.01 ± 4.07	18.03 ± 4.04	18.00 ± 3.99
DFA	0.95 ± 0.12	0.94 ± 0.13	0.94 ± 0.13	0.94 ± 0.13
MFDFA	0.97 ± 0.12	0.97 ± 0.13	0.96 ± 0.14	0.96 ± 0.14

Physiological variables (*N* = 33). G1, baseline; G2, early simulated plateau; G3, later simulated plateau; and G4, recovery.

* *p* < 0.05;

*** *p* < 0.001 for difference with G1.

† *p* < 0.05.

To show the dynamic change of HRV parameters during the experiment, four featured HRV measures from different perspectives are selected and illustrated, including RMSSD in time-domain, LF-HF-ratio in frequency-domain, SD1/SD2-ratio based classical chaos theory, and GI based HRA in the non-linear analysis. Figure 2 shows the box-plot distribution of characteristics and central 95% intervals for all subjects in the experiment’s longitudinal groups.

## Discussion

### Significance of HRV Changes

This prospective study focused on longitudinal changes in healthy young college students’ HRV acutely transitioned to a simulated high altitude hypoxia environment. These results indicate that: (1) Acute exposure of subjects to low-pressure hypoxia instead of normobaric normoxia results in some significant HRV metrics effects. Sympathetic activation seems more pronounced in the simulated altitude environment, and heart rate increases significantly. (2) The subjects’ non-linear HRV characteristics did not change significantly in the controlled hypobaric and hypoxia environment, and the characteristics recovered quickly after exiting the oxygen chamber. (3) RCMSE presents slight differences in multiscale physiological complexity between groups more intuitively than other complexity features, such as ApEn and SampEn. Participants acutely exposed to hypobaria hypoxia for up to 1 h had significant differences in individual complexity before and after each half, which may be related to their stress adaptation capacity. Our study is more comprehensive than previous studies because of a more rigorous experimental paradigm and prolonged acute exposure to hypobaria hypoxia, and analysis of longitudinal changes in HRV.

Heart rate variability time-domain characteristics are a relatively intuitive way to obtain useful information, which analysis the RR interval sequences with a certain degree of randomness using traditional statistical methods. The PSD visualizes the distribution and phase variation of the ECG signal in each frequency band. Simultaneously, the Poincaré plot is a multidimensional “spatially structured” cross-sectional diagram with non-linear chaotic properties (Ciccone et al., 2017) used to observe and study the evolution of non-linear systems. The latter plots the Cartesian plane’s distribution using the values of adjacent RR intervals as coordinates, thus reflecting the distribution of adjoining RR interval time series in phase space. It can visually demonstrate the HRV information between heartbeats (Brennan et al., 2001). In this paper, the real-time dynamic fluctuations and the overall trend of heart rate, PSD, and Poincaré plot throughout the simulated plateau experiment are shown in Figures 1, 2.

The combined real-time HRV trend graph and PSD show that the subjects’ heart rate in the controlled hypobaric and hypoxia environment showed an increasing trend, with a more pronounced bimodal peak in the low-frequency band and a slightly higher power spectrum high-frequency band. Sympathetic activation can explain the higher heart rate trend, LF/HF ratio spectral measurements, and cardiac sympathetic-vagal balance index. It can also cause a general decrease in heart rate parasympathetic modulation quantified by the reduced HF and LF powers.

These spectral changes are in line with those repeatedly reported on the acute autonomic effects after an ascent at high altitude (Hughson et al., 1994; Perini et al., 1996; Saito et al., 2005; Chen et al., 2008; Faini et al., 2019) or a simulated altitude of 3,600 m asl in a hypobaric/hypoxic chamber. Similarly, Boos et al. (2018) found an increase in LF power but without a change in HF power at relatively lower altitudes (3,000 m asl). However, differently from our experimental results, Chen et al. (2008) collected recordings during sleep, including apnea or periodic ventilation. The sleep state is also considered a parasympathetically dominated activity; the state of the subjects influences the results of the experiment, so the conclusion does not apply to our concern. Nevertheless, most studies have concluded that parasympathetic activity decreases at sudden altitude increases (Hughson et al., 1994; Perini et al., 1996; Saito et al., 2005), even more so at 5,500 m asl (Aebi et al., 2020). In addition, we also found that the frequency-domain parameters had relatively high variability within participants, which is consistent with other studies, may imply that more comprehensive assessment metrics often need to be explored in HRV analysis.

Participants had a “comet-like” appearance at baseline (Figures 3E–H), with a short RR interval corresponding to the head and a relatively small distribution area, indicating a small degree of arrhythmia when the heart rate is fast. The more extended distribution area of the scatter along the contour (main diagonal, the line crossing the origin at 45°) indicates an extensive range of heart rate changes, consistent with normal subjects’ heart rate alteration pattern (Satti et al., 2019). It is generally accepted that the scatter plot fitted ellipse’s long axis represents the overall variability of heart rate and the short axis of the ellipse represents the difference between adjacent RR intervals, expressing changes in transient power and reflecting parasympathetic activity. During the first 30 min in the hyperbaric chamber, the heart rate increased abruptly with a dense scatter distribution, a broader baseline, and a more flat and narrow fitted ellipse. Furthermore, there were still large heart rate fluctuations during the recovery phase after the oxygen chamber experiment compared with the baseline. It has been pointed out that all these traditional quantitative indicators are correlated with the time and frequency domain indicators of HRV (Brennan et al., 2001; Ciccone et al., 2017). For example, the long and short axis measures SD1 and SD2 are directly associated with the time domain indicators SDSD and SDNN, which can be expressed as follows:


$$SD1^{2}=\frac{1}{2}SDSD^{2}$$

$$SD2^{2}=2SDNN^{2}-\frac{1}{2}SDSD^{2}$$

Thus, our findings suggest that the short elliptical axis (SD1) standard deviation was significantly lower in the controlled hypobaric and hypoxia environment subjects than normobaric pressure (Table 5), implying a decrease in parasympathetic activity, in other words, a predominance of sympathetic control. This finding is consistent with the study by Chen et al. (2020).

In addition to the time-frequency domain characteristics of HRV, its non-linear analysis gradually becomes a hot spot for research. And this study mainly includes classical chaos theory, power-law analysis, and information-theory-based feature calculation methods (Table 5). The calculation method of non-linear features is usually to reconstruct the phase space by the delayed embedding of HRV time series and study the properties of attractors in the phase space to obtain the relevant kinetic properties of heart rate modulation.

The MSE proposed by Costa et al. (2005b) is the original and effective algorithm for measuring multiscale complexity in physiological signals, such as RR intervals. Compared with MSE, RCMSE can reduce the probability of inducing fuzzy entropy, which is more precise and reliable for short term time series. We presented the RCMSE measured the cardiovascular system’s complexity, instead of the traditional MSE method, in Tables 4 and its overall changes during the four scenarios within 160 min in Figure 4. Stays at high altitudes induce alterations in cardiovascular control.

It is easy to see that the subjects’ complexity was significantly lower in the hypobaria hypoxia environment at all 20 different scales relative to the baseline level. Factors commonly trigger sympathetic-parasympathetic balance, such as postural changes, strenuous activity, or pathological changes (Porta et al., 2012, 2015; Cui et al., 2020). This decreases the SampEn of heart rate and thus significantly reduces the plateau MSE (Table 5), which may also result from sympathetic activation caused by plateau hypoxia. This conclusion is supported by several studies (Hughson et al., 1994; Perini et al., 1996; Saito et al., 2005) in which the balance of the ANS shifts toward relatively low parasympathetic and high sympathetic activity at high altitudes. Coherently with our result, a decrease of the heart-rate SampEn was observed during a real high-altitude environment (Saito et al., 2005; Boos et al., 2018; Faini et al., 2019) and in a simulated high altitude (Zhang et al., 2015). However, Vigo et al. (2010) reported the opposite trend in a low-pressure chamber experiment simulating an altitude of 8,230 m asl, reflecting a rapid activation of defensive autonomic responses induced by the subjects’ sudden exposure to an extreme abnormal environment.

Significant differences in the corresponding complexity indices between the G2 and G3 groups (first and second half hours of the low-pressure phase) are marked in Figure 4 using stars. Further observation of the RCMSE curves for the G2 and G3 groups reveals that the body enters a stressful environment (e.g., a higher altitude environment). And sympathetic activity continues to increase, while parasympathetic decreases, HRV complexity is lower. The body’s ability to adapt to complex environments is diminished, and the findings remain consistent with Faini et al. (2019). Further, our results show that the differences in complexity index (CI) of RCMSE with longitudinal changes over time are mainly concentrated in the low-frequency band and hardly extend to larger scales (e.g., CI scales factor > 8). This may imply that cardiovascular complexity reduction primarily affects faster components, possibly associated with respiratory ventilation, while cardiac complexity is preserved at longer scales.

Besides, we considered the intrinsic complexity that physiological systems have. In healthy states, these systems work in a non-equilibrium state (Costa et al., 2005a), and asymmetry is a fundamental property of non-equilibrium systems (Chialvo and Millonas, 1995; Prigogine and Antoniou, 1999). Heart rate regulatory systems under the ANS control show an inevitable variability (as shown in the heart rate timing diagram shown in Figure 4). And macroscopically, the heart rate (or RR interval) is usually in an alternating pattern of rising and falling with each other. Therefore, studies have used HRA to characterize this prevalent phenomenon in healthy heart physiological systems, such as GI, SI, AI, and PI. According to the literature, approximately 80% of healthy individuals display HRA, and that healthy physiological systems exhibit the highest HRA at rest (Piskorski and Guzik, 2007). HRA is considered the state shown by healthy physiological systems (Yan et al., 2017), and a decrease in HRA often indicates some pathological condition.

### Main Findings

This paper has some common points with the above-presented studies on related topics. In fact, these studies have discussed the changes in the classical parameters of HRV in humans in a hypobaric hypoxic environment, although some disagreement remains on certain issues. In addition, several studies on the effects of high altitude and hypoxic environments on human ANS activity already existed earlier. Hansen et al. measured sympathetic nerve activity directly by peroneal microneurography in eight healthy volunteers after 4 weeks at an altitude of 5,260 m and at sea level (Hansen and Sander, 2003). However, the authors are more concerned with sympathetic activation during prolonged exposure to hypoxia. Many studies demonstrate the ability of acute hypoxia to activate sympathetic nerves in muscles through using direct microneurographic recordings of sympathetic discharge to the skeletal muscle vascular bed (Saito et al., 1988; Rowell et al., 1989; Somers et al., 1989, 1991; Leuenberger et al., 1991; Morgan et al., 1995; Duplain et al., 1999; Hansen et al., 2000; Xie et al., 2001). Although resting levels of muscle sympathetic nerve activity are well correlated with norepinephrine spillover in the heart and kidney (Wallin et al., 1992, 1996), we cannot extrapolate their findings concerning effects of chemical stimuli on muscle sympathetic nerve activity to other organs or vascular beds (Xie et al., 2001).

Nevertheless, this work adds some new elements to the past studies. These include but are not limited to:

1. We are the first study to focus on the utility of all HRV parameters mentioned so far to change during acute stress states in humans. It also focuses on the physiological significance and change process of the novel non-linear parameters, which can provide richer evidence to support the relationship between HRV and ANS regulation through this experiment.
2. In contrast to direct microneurographic recordings of sympathetic discharge to the skeletal muscle vascular bed, we deploy advanced medical-grade wearable devices to observe a full range of HRV parameters with more flexible application prospects. For example, wearable devices that integrate these HRV parameters can be used to monitor the body’s autonomic activity during high altitude operations, allowing timely response to the health risks of acute hypoxia.
3. The experimental paradigm of this study is more rigorous. Our experiment tracked the changes in HRV parameters throughout the participants’ life, including the baseline phase, the change phase of acute exposure to the hypobaric hypoxic environment, and the recovery phase.
4. This study used a larger and gender-balanced group of participants for the experiment, thus ensuring a more reliable analysis of the relevant parameters and higher robustness of the study results.

### Limitations and Future Work

The present study trial was a prospective study design, and the aim was to reveal the regulatory role of ANS through the process of changes in multimodal parameters of HRV in a population acutely exposed to a hypobaric hypoxic environment. However, despite our strict control of experimental variables, large individual differences were found for individual HRV parameters, especially frequency domain indicators. This problem is also present in previous works (Boardman et al., 2002; Bachler, 2017); we believe that further expansion of the sample size in future studies may help to reduce this bias. In addition, there are still some limitations in the time/frequency domain parameters of HRV, which may also contribute to the divergent results of most existing studies. For example, linear time-domain parameters make it difficult to fully characterize complex cardiac kinetic systems, while frequency-domain parameters are also subject to interference from environmental noise or human motion, which may affect the mapping link between HRV analysis and human ANS regulatory mechanisms.

Moreover, most of the features in the non-linear parameters did not show good differential indicative properties. For example, heart rate fragmentation and HRA-related parameters, due to their complexity in characterizing physiological systems, are less sensitive to physiological alterations than features such as RCMSE that calculate the degree of fine-grained disorganization. More attention should be paid to the value of novel parameters like MSE in the future. Finally, while the present study does assess longitudinal changes, it is important to emphasize that this paper only examines acute changes and that experiments still need to be designed to investigate the adaptive processes that regulate the ANS in response to stress over a longer period.

## Conclusion

The current work simulated a plateau environment to investigate the response and rehabilitation in ANS of healthy young subjects. To the best of our knowledge, this paper is the first longitudinal study with “normxia-hypoxia-normxia” as an experimental paradigm and then analyzes the complete time-series change process of the HRV. This study’s findings agree with common sense and previously published results. To be more specific, our work demonstrates that increased heartbeat sequence complexity, significant differences in some time-frequency domain HRV features, the significantly lower overall complexity of RCMSE, and dominant sympathetic activity controlling cardiac activity can be observed in the subjects of the experiment when acute exposure to a hypobaric and hypoxic environment. In addition, some supportive evidence has proved that methods in this paper, such as RCMSE analysis, are more useful in detecting minor differences in RR interval series, which promised to be a great approach that can be applied in follow-up suboptimal state detection or stress response studies.

## Data Availability Statement

The datasets presented in this article are not readily available because patient privacy needs to be protected. Requests to access the datasets should be directed to contact the Department of Neurosurgery, First Medical Center of Chinese PLA General Hospital.

## Ethics Statement

The studies involving human participants were reviewed and approved by Ethics Committee of PLA General Hospital. The patients/participants provided their written informed consent to participate in this study. Written informed consent was obtained from the individual(s) for the publication of any potentially identifiable images or data included in this article.

## Author Contributions

CM performed the main data analysis work and wrote the manuscript. HX designed the experiment and revised this manuscript. MY and JH provided experimental facilities. MY, JH, and WY offered helpful clinical suggestions. KL helped to analyze the data. ZZ and JW provided the overall technical guidance and methodological support. All authors contributed to the study design and result interpretation. All authors contributed to the article and approved the submitted version.

## Funding

This work was supported by The National Natural Science Foundation of China (62171471), Beijing Municipal Science and Technology (Z181100001918023), and Big Data Research & Development Project of Chinese PLA General Hospital (2018MBD-08 and 2018MBD-09).

## Conflict of Interest

KL was employed by the company Beijing SensEcho Science & Technology Co., Ltd., Beijing, China.

The remaining authors declare that the research was conducted in the absence of any commercial or financial relationships that could be construed as a potential conflict of interest.

## Publisher’s Note

All claims expressed in this article are solely those of the authors and do not necessarily represent those of their affiliated organizations, or those of the publisher, the editors and the reviewers. Any product that may be evaluated in this article, or claim that may be made by its manufacturer, is not guaranteed or endorsed by the publisher.
